# Impact of ferroptosis-related risk genes on macrophage M1/M2 polarization and prognosis in glioblastoma

**DOI:** 10.3389/fncel.2023.1294029

**Published:** 2024-01-10

**Authors:** Xin Xu, Yue Zhang, Chenlong Liao, Han Zhou, Yiwei Wu, Wenchuan Zhang

**Affiliations:** Department of Neurosurgery, Shanghai Ninth People’s Hospital, School of Medicine, Shanghai Jiao Tong University, Shanghai, China

**Keywords:** glioblastoma, ferroptosis, M1/M2 macrophages, macrophage polarization, prognosis

## Abstract

**Objective:**

To explore the effect impact of ferroptosis on macrophage polarization and patient prognosis in glioblastoma.

**Methods:**

We screened ferroptosis-related risk from the public datasets of primary and recurrent glioblastoma, combined with reported ferroptosis genes, calculated the risk genes among the ferroptosis-related genes using the LASSO Cox regression model, and investigated the relationship between these ferroptosis-related risk genes in the tumor and the spectrum of infiltrating M1/M2 macrophages. Macrophages were analyzed using the CIBERSORTx deconvolution algorithm. Samples from The Cancer Genome Atlas (TCGA), Chinese Glioma Genome Atlas (CGGA) and a single-cell RNA sequencing dataset (GSE84465) were included. The expression levels of ferroptosis-related risk genes and molecular markers of M1 and M2 macrophages were detected by qPCR and western blot.

**Results:**

A total of fourteen ferroptosis-related risk genes were obtained and the patients’ risk scores were calculated. Compared with patients in the low-risk group, patients in the high-risk group had worse prognosis. The M1/M2 macrophage ratio and risk score were negatively correlated, indicating that the tumor microenvironment of glioblastoma in the high-risk group contained more M2 than M1 macrophages. In the single-cell RNA sequencing dataset, the risk score of ferroptosis-related genes in tumor cells was positively correlated with the proportion of high M2 macrophages. The expression of eight ferroptosis-related risk genes was increased in glioblastoma cell, which promoted the polarization of M1 macrophages to M2.

**Conclusion:**

We investigated the fourteen ferroptosis-related risk genes in glioblastoma for the first time, and clarified the impact of ferroptosis-related risk genes on M1/M2 macrophage polarization and patient prognosis.

## 1 Introduction

Glioma is the most common type of primary malignant tumor of the central nervous system and is most likely to occur in adults aged 45–65. Glioblastoma multiforme (GBM) is a representative glioma, accounting for 49.1% of all malignant tumors in central nervous system (CNS) (Chi et al., 2023). The World Health Organization (WHO) grading system classifies gliomas into four grades, of which GBM is classified as grade 4 (Louis et al., 2021). Currently, the treatment of glioma includes surgical resection, immunotherapy, radiotherapy, chemotherapy and novel molecular targeted therapy (Jhaveri et al., 2018; Xu et al., 2020), while GBM patients have been observed to have the shortest median patient survival, with only 5.8% of GBM patients surviving for 5 years (Luo et al., 2022). Despite the recent advances in clinical management, the prognosis of malignant glioma patients remains relatively poor (Fang and Zhang, 2020). Therefore, research into new therapeutic strategies for gliomas, especially for GBM, is still urgently needed.

Ferroptosis is a newly identified form of programmed cell death, distinct from apoptosis, cell necrosis and autophagy, characterized by iron-dependent lipid peroxidation (Chi et al., 2023). Ferroptosis is mainly due to redox imbalance and involves several intracellular biological processes, such as iron metabolism, lipid metabolism, and antioxidant synthesis (Zhao et al., 2022). Induction of ferroptosis may be a novel target for glioma treatment, and ferroptosis-related processes are associated with chemo- and radioresistance in glioma (Shi et al., 2022).

Tumor-associated macrophages (TAMs) and ferroptosis are coordinately regulated, and thus co-regulate response to tumor immunotherapy (Zhou et al., 2022). As an essential component of the tumor immune microenvironment, TAMs are a key factor in the efficacy of tumor immunotherapy. Two distinct cell polarizations of macrophages have opposing functions in tumor progression. The classic phenotype, namely M1 macrophages, function in antigen presentation, induce inflammatory responses, scavenge pathogenic microorganisms, and exert antineoplastic effects within the tumor immune microenvironment. In contrast, the alternative phenotype, namely M2 macrophages are able to limit the inflammatory response and contribute to tumor progression by stimulating proliferation, angiogenesis, and metastasis (Mantovani et al., 2017). Notably, M2-like TAMs are highly associated with therapeutic resistance and are regarded as providing a barrier to effective tumor immunotherapy (Zhou et al., 2022).

In this study, we screened fourteen ferroptosis-related risk genes and calculated risk scores for patients with glioma based on gene expression data and clinicopathologic information collected from glioma patient databases, and investigated the relationship between the expression of these genes and clinicopathologic factors as well as macrophage infiltration of glioma to explore the role of ferroptosis-related genes in regulating ferroptosis and macrophage polarization in GBM.

## 2 Materials and methods

### 2.1 Data source and acquisition

In this study, a total of 1,528 glioma patient samples with corresponding clinical information were obtained from two primary databases: the Cancer Genome Atlas (TCGA)^1^ and the Chinese Glioma Genome Atlas (CGGA).^2^ Subsequently, samples lacking essential clinical information were filtered out, ensuring a cohort consisting exclusively of patient samples with complete clinical data for analysis.

For single-cell RNA sequencing analysis, a Gene Expression Omnibus (GEO) dataset (GSE84465) utilizing the chip platform GPL18573 [Illumina NextSeq 500 (Homo sapiens)] was employed, recognized as a widely adopted platform for transcriptome analysis.

### 2.2 Identification of differentially expressed genes (DEGs) in the TCGA datasets

**FIGURE 1:**
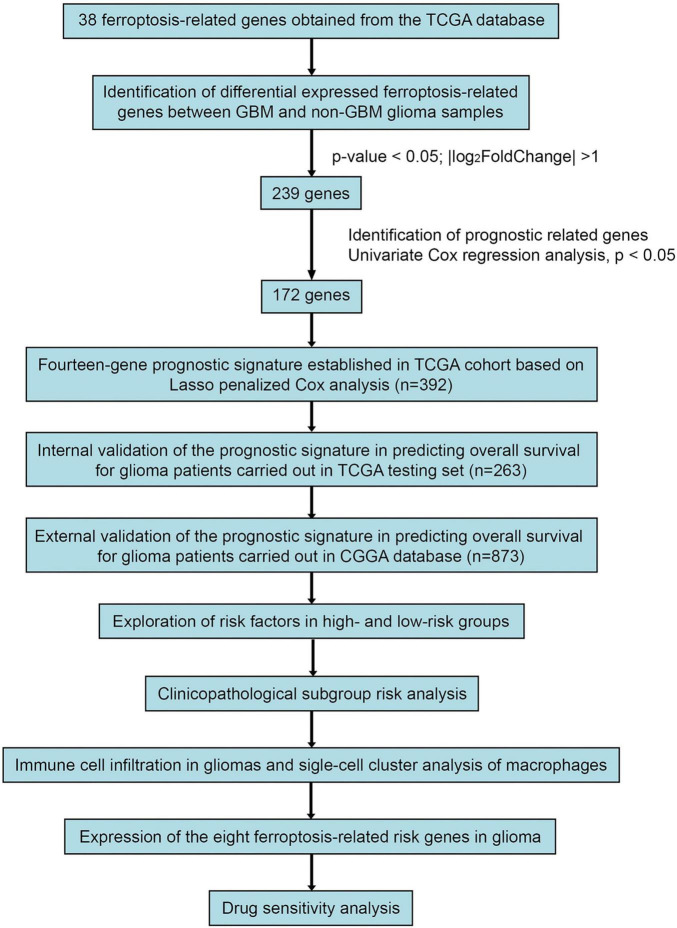
Study Workflow.

Based on the ferroptosis-related gene sets obtained from MSigDB,^3^ we extracted data on 382 ferroptosis-related genes from the TCGA database. The “Limma” R package was used to identify DEGs between GBM and non-GBM glioma tissues, employing a threshold value was set as | log_2_FoldChange (FC)| > 1 and adjusted *p*-value < 0.05. Subsequently, 655 patient samples from the TCGA database were segregated into training and testing sets at a ratio of 3:2. Figure 1 presents the workflow of this study. The GSE84465 data encompassed single-cell RNA-seq analysis of infiltrating neoplastic human GBM cells (Darmanis et al., 2017), as part of the Brain Immune Atlas incorporating single-cell RNA sequencing datasets from the Movahedi lab across multiple publications (Van Hove et al., 2019; Shemer et al., 2020; Pombo Antunes et al., 2021). We adopted and analyzed the profile generated by Pombo et al., elucidating myeloid cell profiles in GBM.

### 2.3 Bioinformatic analysis

The common differential expression genes were obtained using the “venn” package. The relationship between ferroptosis-related genes and patients’ overall survival (OS) was evaluated by univariate Cox regression analysis using R software. Then, the “glmnet” package was used to perform the LASSO Cox regression model (with the penalty parameter estimated by 10-fold cross-validation) (Pan et al., 2020). A risk score formula based on the expression levels of ferroptosis-related genes for OS prediction was created, where the risk score was (3.74863 × expression level of NRP2) + (2.223197 × expression level of PARVA) + (0.626251 × expression level of LAMA4) + (0.463306 × expression level of EHD2) + (0.455573 × expression level of ANTXR2) + (0.074203 × expression level of DPYSL3)–(2.37796 × expression level of SDC3)–(2.47335 × MTMR12)–(2.71575 × expression level of SH3PXD2A). The risk scores were determined for all patients included in this study, and the median value was selected as the cut-off value to divide patients into high-risk and low-risk groups. Meanwhile, GBM gene expression data were used to calculate the relative proportion of 22 different genotypes in immune infiltrating cells using the CIBERSORT algorithm.

### 2.4 Cell clustering and cell-type annotation

Highly variable genes were extracted to perform principal component analysis (PCA), and the top 30 significant principal components were used for cluster analysis. Clusters were visualized using Uniform Manifold Approximation and Projection (UMAP) and t-Distributed Stochastic Neighbor Embedding (t-SNE). Genes specifically expressed in each cluster were identified using Seurat’s FindAllMarkers function. Significance of differences in gene expression was determined using the Wilcoxon rank-sum test with Bonferroni’s correction, and cell types were manually annotated based on the cluster markers. Macrophage markers were obtained by reviewing previously published literature and the CellMarker website.^4^

### 2.5 Quantitative real-time polymerase chain reaction (qPCR) and western blot analyses

For RNA extraction and quantitative real-time PCR, total RNA was extracted from glioma cells using TRIzol reagent (Invitrogen, Carlsbad, CA, USA) according to the manufacturer’s protocol. The concentration of the isolated RNA was measured, and complementary cDNA was transcribed using the RevertAidTM First Strand cDNA Synthesis Kit (Takara Bio, Shiga, Japan). The RNA content was measured according to the instructions of the Invitrogen RNA extraction kit. And a reverse transcription kit was used to reverse transcribe the secretory RNA into cDNA. A 20 μL reaction system was used for PCR amplification and detection by BIO-RAD fluorescent real-time PCR. The primer sequences employed for PCR amplification are detailed in the Supplementary material accompanying this study.

For Western blot analysis, cells were lysed with radioimmunoprecipitation assay lysis buffer (RIPA, Beyotime, Shanghai, China) mixed with a phenylmethanesulfonyl fluoride protease inhibitor at a ratio of 100:1 (Beyotime, Shanghai, China). Protein concentrations were determined using a bicinchoninic acid protein assay kit (Beyotime, Shanghai, China). A 30 μg protein sample was used for 10% sodium dodecyl sulfate polyacrylamide gel electrophoresis. After electrophoresis, the protein sample was transferred to PVDF membrane (Millipore) by the wet conversion method, and the skimmed milk powder was sealed. After overnight incubation with primary antibodies at 4°C, TBS buffer was added to wash the membrane, and then it was incubated with secondary antibodies (Abcam) for 2 h at room temperature. TBST buffer was used for washing, and ECL reagent (Beyotime, Shanghai, China) was used for band visualization. The expression of the target protein was equal to the grayscale value of the target band/the grayscale value of the GAPDH band.

### 2.6 Statistical analysis

The statistical analysis was conducted using SPSS version 23.0 (SPSS Inc., Chicago, IL, USA) and R software version R-4.0.2 (The R Foundation for Statistical Computing, Vienna, Austria) on a Windows platform. Baseline demographic characteristics, hazard ratios of risk factors, and mRNA expression levels were presented as mean ± standard deviation (SD). To identify independent prognostic factors for overall survival (OS), both univariate and multivariate Cox regression models were employed. Hazard ratios (HRs) along with their 95% confidence intervals (CIs) were calculated. The association between variables and OS was assessed using the Kaplan-Meier method, and survival curves were compared using the log-rank test. Additionally, statistical significance between different groups was evaluated using chi-square tests, Student’s *t*-tests, and analysis of variance (ANOVA). A significance threshold of *P* < 0.05 was set to determine statistical significance.

## 3 Results

### 3.1 Identification of ferroptosis-related risk genes

A total of 655 patient samples were stratified into a training set and a testing set using a 3:2 ratio. Among these, 392 patient samples were allocated to the training set, while 263 patient samples comprised the testing set. The classification based on patients’ pathologic types involved assigning patient samples with a WHO classification of grade 4 to the GBM group, whereas patient samples falling within WHO grades 2–3 were designated to the non-GBM group. Comprehensive baseline demographic characteristics of the TCGA dataset samples can be found in Table 1.

**TABLE 1:** Baseline demographic characteristic (TCGA).

Variables	GBM testing set (*n* = 66)	GBM training set (*n* = 84)	Non-GBM testing set (*n* = 197)	Non-GBM training set (*n* = 308)
**Age, n (%)**				
<45	5 (7.58%)	12 (14.29%)	117 (59.39%)	180 (58.44%)
≥45	61 (92.42%)	72 (85.71%)	80 (40.61%)	128 (41.56%)
**Gender, n (%)**				
Female	26 (39.39%)	24 (28.57%)	92 (46.70%)	134 (43.51%)
Male	40 (60.61%)	60 (71.43%)	105 (53.30%)	174 (56.49%)
**Race (%)**				
Asian	1 (1.52%)	4 (4.76%)	5 (2.54%)	4 (1.30%)
Black or African American	6 (9.09%)	4 (4.76%)	7 (3.55%)	14 (4.55%)
White	59 (89.39%)	75 (89.29%)	181 (91.88%)	284 (92.21%)
Not reported	0 (0.00%)	1 (1.19%)	4 (2.03%)	6 (1.95%)
**Grade (%)**				
G2	0 (0.00%)	0 (0.00%)	90 (45.69%)	154 (50.00%)
G3	0 (0.00%)	0 (0.00%)	107 (54.31%)	154 (50.00%)
G4	66 (100.00%)	84 (100.00%)	0 (0.00%)	0 (0.00%)
**Sample type (%)**				
Primary tumor	62 (93.94%)	79 (94.05%)	194 (98.48%)	303 (98.38%)
Recurrent tumor	4 (6.06%)	5 (5.95%)	3 (1.52%)	5 (1.62%)
**Primary site (%)**				
Brain, NOS	66 (100.00%)	84 (100.00%)	4 (2.03%)	4 (1.30%)
Frontal lobe	–	–	116 (58.88%)	181 (58.77%)
Occipital lobe	–	–	2 (1.02%)	6 (1.95%)
Parietal lobe	–	–	24 (12.18%)	22 (7.14%)
Posterior fossa, cerebellum	–	–	1 (0.51%)	1 (0.32%)
Temporal lobe	–	–	50 (25.38%)	94 (30.52%)
**Laterality (%)**				
Left	–	–	99 (50.25%)	148 (48.05%)
Midline	–	–	1 (0.51%)	4 (1.30%)
Right	–	–	97 (49.24%)	156 (50.65%)
**First presenting symptom (%)**				
Headaches	–	–	46 (23.35%)	57 (18.51%)
Mental status changes	–	–	13 (6.60%)	26 (8.44%)
Motor/movement changes	–	–	18 (9.14%)	19 (6.17%)
Seizures	–	–	83 (42.13%)	162 (52.60%)
Sensory changes	–	–	8 (4.06%)	9 (2.92%)
Visual changes	–	–	5 (2.54%)	6 (1.95%)
Unknown	–	–	24 (12.18%)	29 (9.42%)
**Headache history (%)**				
No	–	–	106 (53.81%)	183 (59.42%)
Yes	–	–	69 (35.03%)	98 (31.82%)
Unknown	–	–	22 (11.17%)	27 (8.77%)
**Seizure history (%)**				
No	–	–	76 (38.58%)	98 (31.82%)
Yes	–	–	108 (54.82%)	190 (61.69%)
Unknown	–	–	13 (6.60%)	20 (6.49%)
**Mental status changes (%)**				
No	–	–	124 (62.94%)	214 (69.48%)
Yes	–	–	49 (24.87%)	64 (20.78%)
Unknown	–	–	24 (12.18%)	30 (9.74%)
**Sensory changes (%)**				
No	–	–	138 (70.05%)	239 (77.60%)
Yes	–	–	31 (15.74%)	37 (12.01%)
Unknown	–	–	28 (14.21%)	32 (10.39%)
**Visual changes (%)**				
No	–	–	145 (73.60%)	242 (78.57%)
Yes	–	–	26 (13.20%)	37 (12.02%)
Unknown	–	–	26 (13.20%)	29 (9.42%)
**Neoadjuvant treatment (%)**				
No	66 (100.00%)	84 (100.00%)	194 (98.48%)	308 (100.00%)
Yes	0 (0.00%)	0 (0.00%)	3 (1.52%)	0 (0.00%)
**Radiation therapy (%)**				
No	9 (13.64%)	12 (14.29%)	59 (29.95%)	107 (34.74%)
Yes	57 (86.36%)	72 (85.71%)	112 (56.85%)	161 (52.27%)
Unknown	0 (0.00%)	0 (0.00%)	26 (13.20%)	40 (12.99%)
**New tumor event after initial treatment (%)**				
No	–	–	107 (54.31%)	163 (52.92%)
Yes	–	–	48 (24.37%)	82 (26.62%)
Unknown	–	–	42 (21.32%)	63 (20.45%)

Furthermore, the testing set from the TCGA dataset served as internal validation, while an independent validation was performed using 873 patient samples from the CGGA dataset. Similar categorization by pathology into GBM and non-GBM groups was undertaken for the CGGA dataset, with respective baseline demographics outlined in Table 2.

**TABLE 2:** Baseline demographic characteristic (CGGA).

Variables	GBM (*n* = 343)	Non-GBM (*n* = 530)	*P*-value
**Age, *n* (%)**			**<0.001**
<45	125 (36.44%)	356 (67.17%)	
≥45	218 (63.56%)	174 (32.83%)	
**Gender, *n* (%)**			**0.317**
Female	136 (39.65%)	224 (42.26%)	
Male	207 (60.35%)	306 (57.74%)	
**Chemo status (%)**			**<0.001**
Yes	283 (82.51%)	342 (64.53%)	
No	60 (17.49%)	188 (35.47%)	
**Radio status (%)**			**0.193**
Yes	280 (81.63%)	412 (77.74%)	
No	63 (18.37%)	118 (22.26%)	
**PRS type (%)**			**<0.001**
Primary	203 (59.18%)	362 (68.30%)	
Recurrent	112 (32.65%)	168 (31.70%)	
Secondary	28 (8.16%)	0 (0.00%)	
**Grade (%)**			**<0.001**
G2	0 (0.00%)	242 (45.66%)	
G3	0 (0.00%)	288 (54.34%)	
G4	343 (100.00%)	0 (0.00%)	
**IDH mutation status (%)**			**<0.001**
Mutant	80 (23.32%)	399 (75.28%)	
Wildtype	263 (76.68%)	131 (24.72%)	

Bold values indicate the *P* < 0.05 to emphasize their statistical significance.

Differential gene expression analysis between the GBM and non-GBM groups within both the training and testing sets revealed 239 commonly differentially expressed genes. Subsequently, 172 genes exhibiting significance (*p*-values < 0.05) were identified following univariate Cox regression analysis. To assess the impact on OS among glioma patients, a Lasso Cox model was constructed to screen for ferroptosis-related risk genes, culminating in the identification of fourteen significant genes (Supplementary Figures 1A, B and Supplementary Table 1).

### 3.2 Identification and verification of viability of fourteen ferroptosis-related risk genes

**FIGURE 2:**
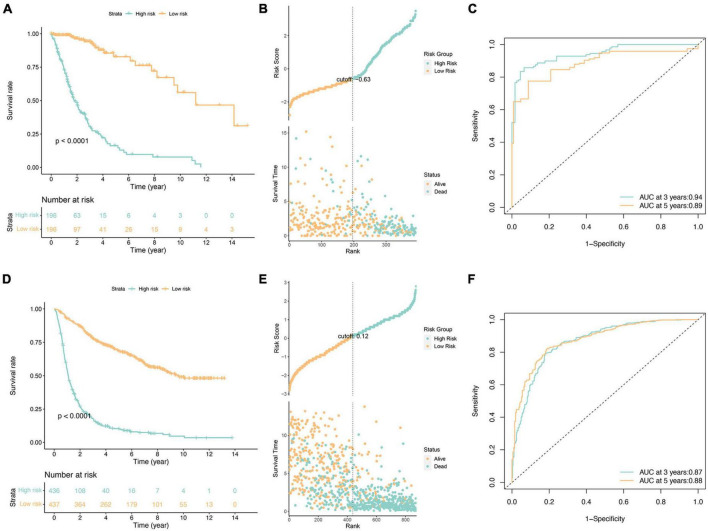
Survival analysis of glioma patients in the TCGA cohorts **(A–C)** and independent validation in the CGGA cohorts **(D–F)**. **(A,D)** Kaplan-Meier survival analysis, **(B,E)** risk score by the fourteen ferroptosis-related risk genes, patients’ survival status and time, **(C,F)** time-dependent ROC curves. The 3 and 5-year AUCs were used to evaluate the prognostic accuracy, and the log-rank test was used to calculate the *p*-value.

Following the application of the Lasso-Cox model, each case was assigned a risk score, leading to the stratification of patients into high-risk and low-risk subgroups based on the median value of the risk score (Figures 2A–C). Notably, the prognosis of the high-risk subgroup exhibited a significantly poorer outcome compared to the low-risk subgroup (Figure 2A). Evaluation of risk score distributions, survival durations, and patient status revealed a potential correlation where lower risk scores appeared associated with longer life expectancies (Figure 2B).

Furthermore, independent validation using the CGGA database (illustrated in Figures 2D–F) substantiated these findings, demonstrating consistent outcomes between datasets. Specifically, the validation reinforced the observation of a notably worse prognosis within the high-risk subgroup, aligning with results obtained from the initial analysis.

### 3.3 Identification and verification of independent risk factors

**FIGURE 3:**
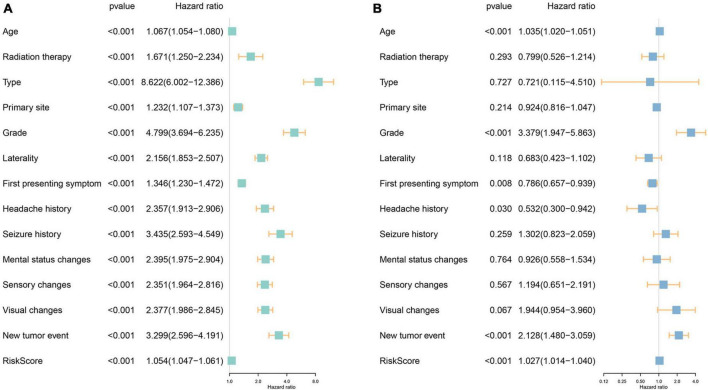
Investigation of the independent prognostic risk factors in glioma patients. Univariate **(A)** and multivariate **(B)** Cox regression analysis of clinicopathologic characteristics were performed, and the hazard ratios (HR) and 95% confidence intervals (CI) were calculated, respectively.

The investigation into the association between fourteen ferroptosis-related risk genes and clinicopathology revealed distinct categorization. AURKA, RRM2, HBA1, CAPG, HSPB1, GDF15, STEAP3, and NNMT were identified as risk genes, suggesting that elevated expression levels might correlate with a poorer prognosis. Conversely, NF2, YY1AP1, XBP1, BLOC1S5, GCLC, and BID were categorized as protective genes, indicative of a potential better prognosis when expressed at higher levels (Supplementary Figure 2). Of particular interest was the notable overexpression of the first eight genes, notably HBA1, GDF15, and NNMT, within patients classified as high-risk. Intriguingly, these genes exhibited significantly heightened expression levels in WHO Grade 4, predominantly among GBM patients, hinting at a possible relationship between their elevated expression and the onset of GBM. Additionally, this subset of genes showed increased expression in patients aged 45 years or older and those with a fatal prognosis, suggesting a potential association with disease occurrence, progression, and an unfavorable prognosis. Subsequent univariate and multivariate Cox regression analyses were conducted to assess clinicopathologic factors (Figures 3A, B). Results identified age, grade, new tumor event, and risk score as independent risk factors. Internal validation (Supplementary Figure 3) and independent validation (Supplementary Figure 4) further supported the significance of age, primary tumor site, grade, headache history, new tumor event, and risk score as independent risk factors, particularly within the high-risk group.

### 3.4 Clinicopathological subgroup risk analysis

**FIGURE 4:**
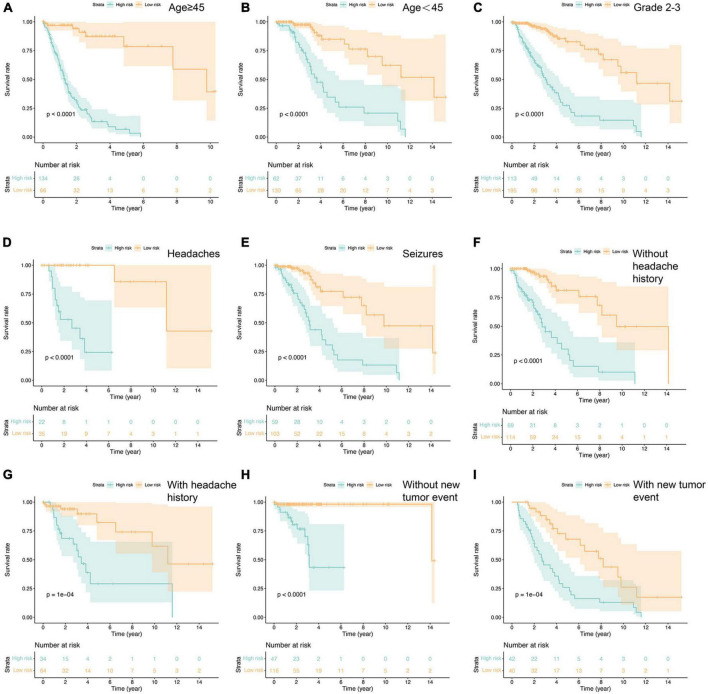
Prognostic analysis in the subtypes with each independent risk factor group **(A–I)**. *P*-value was calculated by log-rank test.

Additionally, we conducted a comprehensive survival analysis for each independent risk factor across clinicopathologic subgroups of glioma patients. The analysis unveiled significantly lower OS rates in the high-risk group compared to the low-risk group within various subgroups of the TCGA cohort. Specifically, this disparity was evident among patients younger than 45 years, aged 45 years or older, graded 2–3 based on the WHO classification, individuals presenting headache and epilepsy as initial symptoms, and those with or without a history of headache and neoplastic conditions (Figure 4). These findings underscore the potential predictive utility of the fourteen ferroptosis-related risk genes for glioma patients across diverse clinical parameters. Notably, the high-risk group exhibited an overwhelming prevalence of WHO grade 4 patients, primarily comprising GBM cases, hinting at the substantially poor prognosis associated with this subgroup. Consequently, no supplementary survival analysis was conducted for this specific subgroup, as the distribution of GBM patients was predominantly confined to the high-risk category.

### 3.5 Analysis of immune cell infiltration in gliomas

**FIGURE 5:**
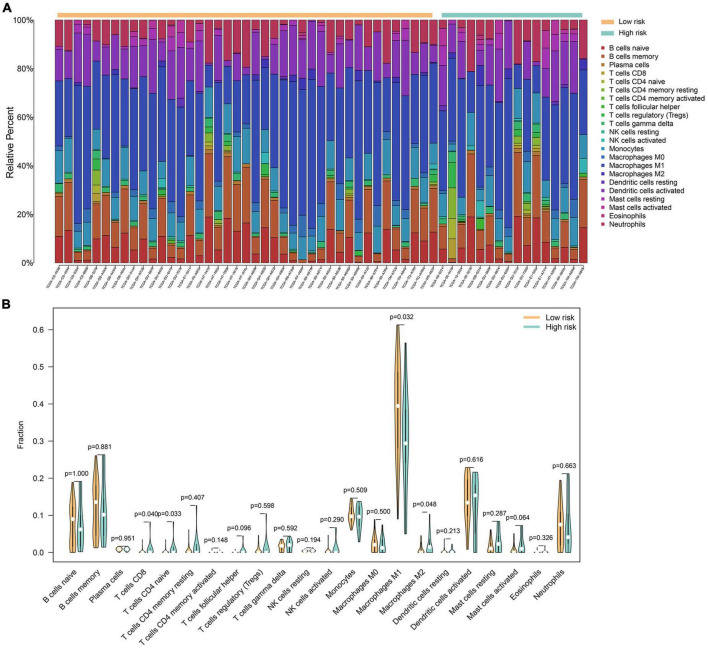
A total of 22 immune cell genotypes infiltration analysis in gliomas. **(A)** The proportions of immune cell subsets in the low- and high-risk groups were analyzed in the TCGA dataset. **(B)** The violin map showed statistical differences between the immune cells of low- and high-risk groups.

The analysis of immune cell infiltration within gliomas revealed distinctive patterns among 22 immune cell genotypes. Notably, a discernible trend emerged wherein the low-risk group exhibited lower expression levels of macrophage M1, while conversely demonstrating higher expression levels of macrophage M2. These differences were statistically significant with *p*-values below 0.05, implying a potential association between macrophage M2 polarization and a negative impact on glioma prognosis. Conversely, the presence of higher macrophage M1 levels hinted at a potential protective factor associated with this immune cell subtype. Moreover, within the high-risk group, there was notable elevation observed in the expression levels of CD8 + T cells and CD4 naïve T cells, both displaying statistically significant differences. These findings bear implications for subsequent studies, suggesting a necessity for further investigation into the role and implications of these immune cell subtypes in the prognosis and progression of gliomas (Figures 5A, B).

### 3.6 Single-cell cluster analysis of macrophages

**FIGURE 6:**
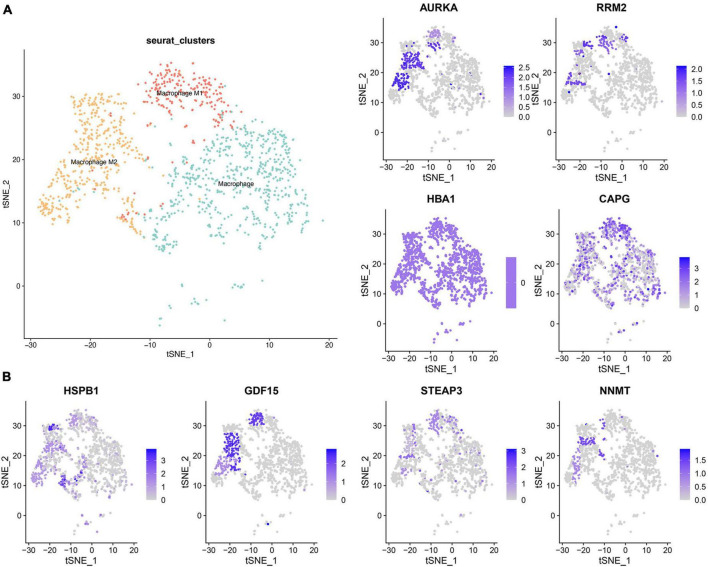
Single cell cluster analysis of macrophages in GBM. **(A)** The three identified macrophage classes in GBM. **(B)** The expression of eight ferroptosis-related risk genes in macrophage clusters.

Distinguishing variations in the distribution of M1 and M2 macrophages between high and low-risk groups prompted an in-depth investigation. To delve deeper, we employed the GSE84465 database, focusing on cell alterations in GBM using the “seurat” R package to scrutinize cell types in human primary GBM samples (Supplementary Figure 5). Leveraging prior literature and the CellMarker website (see text footnote 4), we identified three distinct clusters of macrophages (Figure 6) and compiled a set of macrophage markers (Supplementary Table 2).

Subsequently, the examination of the eight ferroptosis-related risk genes, notably overexpressed in the high-risk group, led to an intriguing observation. It revealed that AURKA, HSPB1, and NNMT exhibited high expression levels in M2 macrophages. Meanwhile, RRM2, GDF15, and STEAP13 were expressed in both M1 and M2, and CAPG demonstrated high expression across all three macrophage clusters. This confluence of findings suggests distinctive patterns of gene expression within specific macrophage clusters, shedding light on potential correlations between ferroptosis-related genes and distinct macrophage phenotypes.

### 3.7 Expression of the eight ferroptosis-related risk genes in glioma

**FIGURE 7:**
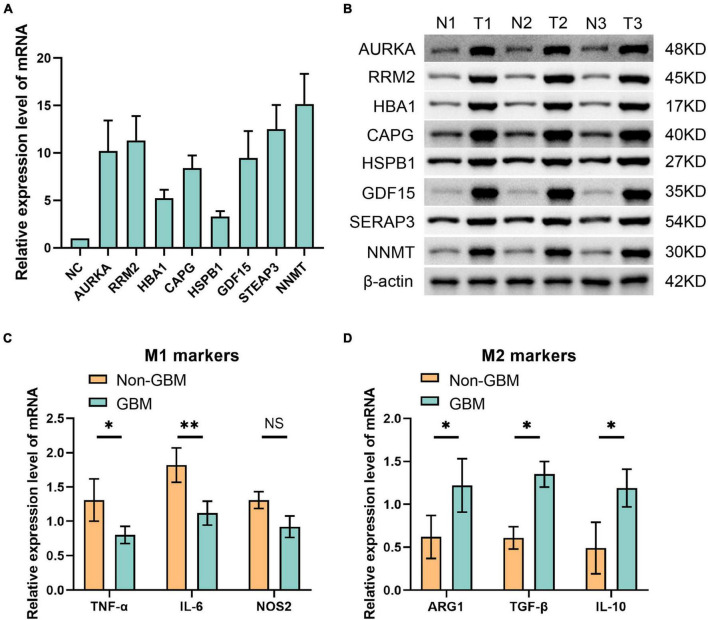
Eight ferroptosis-related genes were detected by panel **(A)** qPCR at mRNA level and **(B)** Western blot at protein level. **(C)** qPCR was used to detect the molecular markers of M1 macrophages in non GBM and GBM groups. **(D)** The molecular markers of M2 macrophages in non GBM and GBM groups were detected by qPCR. *p < 0.05 and **p < 0.01.

In alignment with our earlier observations, eight ferroptosis-related risk genes—AURKA, RRM2, HBA1, CAPG, HSPB1, GDF15, STEAP3, and NNMT—displayed heightened expression levels within the GBM group and notably in the macrophage M2 phenotype. Consequently, we conducted a comprehensive assessment of these genes’ expression within gliomas (Figures 7A–D). Our RT-PCR analysis of the mRNA levels pertaining to these eight ferroptosis-related genes unveiled a noticeable increase in their relative expression levels across all genes assessed (Figure 7A). Further, employing Western blot analysis targeting the relevant proteins confirmed significantly elevated expression levels of all eight ferroptosis-related genes in the glioma group compared to the non-tumor group (Figure 7B). Moreover, our investigation extended to examining the mRNA expression levels of M1 and M2 macrophage markers in both GBM and non-GBM glioma samples. The results from RT-PCR demonstrated a notable upsurge in the expression of two M1 macrophage markers—TNF-α and IL-6—in non-GBM gliomas. Conversely, the expression levels of three M2 macrophage markers—ARG1, TGF-β, and IL-10—were notably increased in GBM samples (Figures 7C, D). These findings elucidate distinct patterns of gene expression within specific macrophage subtypes and their association with ferroptosis-related genes in gliomas.

### 3.8 Drug sensitivity analysis of the eight ferroptosis-related genes

**FIGURE 8:**
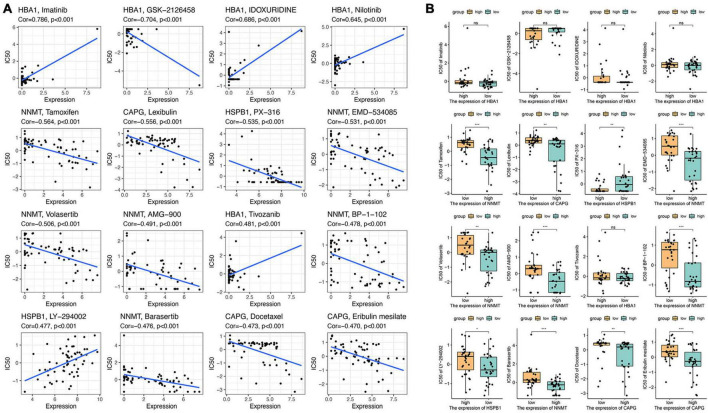
Drug sensitivity analysis of eight ferroptosis-related genes. **(A)** The relationship between gene expression and the drug sensitivity for the 16 selected drugs. **(B)** The relationship between gene expression and drug sensitivities in low- and high-risk groups. NS means No significant, *p < 0.05, **p < 0.01, and ***p < 0.001.

The connection between macrophage-ferroptosis interplay and its implications on immune tolerance and drug resistance in tumors has been highlighted in research (Zhou et al., 2022). The substantial challenge in GBM prognosis is primarily attributed to the resistance of cancer cells to drugs. Addressing this through the modulation of the ferroptosis pathway might present an effective strategy. Considering the heightened expression of the aforementioned eight ferroptosis-related genes in GBM, they potentially offer a promising avenue for GBM pharmacotherapy. To explore this prospect, we specifically targeted these eight genes and conducted a screening of 16 drugs highly correlated with them. We focused on drugs approved by the US Food and Drug Administration (FDA) or undergoing clinical trials sanctioned by the FDA (Figure 8A). Notably, our analysis unveiled intricate correlations: the expression of HSPB1 displayed a positive correlation with LY-294002 sensitivity and a negative correlation with PX-316 sensitivity. Meanwhile, NNMT expression demonstrated a negative correlation with drugs like tamoxifen, barasertib, EMD-534085, volasertib, AMG-900, and BP-1-102. Additionally, CAPG expression exhibited negative associations with drugs like lexibulin, docetaxel, and eribulin mesilate. Further, we analyzed drug sensitivity concerning gene expression levels within both high-risk and low-risk groups (Figure 8B). These findings underscore the potential for targeting specific drugs correlated with ferroptosis-related genes, offering insights into therapeutic interventions for GBM.

## 4 Discussion

In this study, we screened fourteen ferroptosis-related risk genes and calculated risk scores for patients with glioma based on gene expression data and clinicopathological information collected from databases. We found that patients in the high-risk group had a significantly worse prognosis than patients in the low-risk group. And in studying the relationship between the fourteen ferroptosis-related risk genes and clinicopathological factors, eight genes, AURKA, RRM2, HBA1, CAPG, HSPB1, GDF15, STEAP3, and NNMT, were significantly highly expressed in the high-risk group. Independent clinicopathologic risk factors were also validated. In the analysis of immune cell infiltration in gliomas, we found that M2 macrophages outnumbered M1 macrophages in the tumor microenvironment of gliomas in high-risk group, and the three ferroptosis-related risk genes, AURKA, HSPB1, and NNMT, significantly inhibited ferroptosis of glioblastoma cells and promoted the polarization of M1 macrophages to M2. The expression of M2 macrophage markers was also higher in GBM patient samples than in non-GBM glioma patient samples, while the opposite was true for M1 macrophage markers. We also analyzed the drug sensitivity of 16 drugs in relation to the expression levels of the above eight genes.

Macrophages can have different activation states depending on the stimuli they encounter. M1 and M2 are two polarized phenotypes of macrophages that have different functions and characteristics (Biswas and Mantovani, 2010). M1 macrophages are usually considered to be pro-inflammatory and anti-tumor, as they can secrete nitric oxide, tumor necrosis factor, and other effector molecules that kill bacteria and intracellular pathogens, as well as participate in inflammation and tissue damage (Mills and Ley, 2014). M2 macrophages are usually considered to be anti-inflammatory and pro-tumor, as they can secrete ornithine, interleukin-10 and other regulatory molecules that promote tissue repair and angiogenesis, as well as participate in immune suppression and tumor development (Gordon and Martinez, 2010; Potas et al., 2015). The switch between M1 and M2 macrophages is a dynamic process influenced by many factors, including cytokines, chemokines, extracellular matrix, etc. Under various physiological and pathological conditions, the ratio and function of M1 and M2 macrophages may change, thereby affecting the outcome of immune responses (Wang et al., 2014; Liu et al., 2021; Zhu et al., 2021, 2023c).

TAMs are important targets for immunotherapy of glioma, and by regulating or inhibiting the activity of TAM, the sensitivity of tumors to drugs such as immune checkpoint inhibitors can be increased, thereby improving the therapeutic effect (Jaiswal et al., 2009). The distribution of M1 and M2 macrophages in gliomas is heterogeneous and correlates with tumor location, progression, and microenvironment. In general, M1 macrophages predominate in the early stages of the tumor and have anti-tumor and pro-inflammatory properties, but as the tumor progresses, they are converted to M2 macrophages, which have pro-tumor and anti-inflammatory properties (Hambardzumyan et al., 2016). M2 macrophages are more prevalent in the peripheral regions of the tumor than in the core, which may be related to the more hypoxic and acidic microenvironment of the core region (Wang et al., 2022; Ren et al., 2023; Zhu et al., 2023b). A study showed that the shape of M1 macrophage is relatively large and rounded, and the M2 macrophage, which is not conducive to antitumor immunity, is elongated in body shape, and that the morphological characteristics have a significant effect on the polarization of massive TAMs. The use of external interventions to control cell shape revealed that elongation of cells promotes the polarization of macrophages toward the M2 genotype (McWhorter et al., 2013). In this study, we found that the proportion of M2 genotype macrophages was higher in GBM compared to the M1 genotype. This also informs our future work, and subsequent studies may include the regulation and morphology of macrophage M1 and M2 polarization.

Ferroptosis exhibits a dichotomous role in glioma progression, acting as a predominant mechanism for programmed cell death while contributing to the construction of an immunosuppressive microenvironment within gliomas. This dual function of ferroptosis is central to tumor cell demise and concurrently fosters an environment conducive to tumor growth, diminishing the host’s anti-tumor immune response (Lu et al., 2022). Recent investigations have highlighted the contrasting impacts of M1 and M2-polarized macrophages on ferroptosis regulation in gliomas. Studies suggest that M1 macrophages can promote ferroptosis in glioma cells, whereas M2 macrophages exhibit an inhibitory effect (Wang et al., 2019b; Zhuo et al., 2022). This interplay underscores the potential significance of modulating M1/M2 polarization as a means to regulate ferroptosis, presenting an intriguing avenue for therapeutic intervention. Strategies targeting M1 polarization to bolster ferroptosis may bolster anti-tumor immunity and impede tumor growth. Conversely, favoring M2 polarization while inhibiting ferroptosis might aid in reducing inflammation and facilitating tissue repair (Szulzewsky et al., 2015). The high malignancy and drug resistance exhibited by gliomas may stem from their adeptness at evading ferroptosis (Huang et al., 2022; Niu et al., 2023; Zhu et al., 2023a). Compounds inducing ferroptosis have been suggested as potential candidates to counteract this evasion mechanism, potentially mitigating enhanced drug resistance in gliomas (Wu et al., 2020). Moreover, ferroptosis triggers a significant accumulation of M2 macrophages in the tumor immune microenvironment, leading to a tolerance in tumor cells against immunotherapy through the interplay between macrophages and ferroptosis (Wang et al., 2019a). This study also explores the correlation between eight ferroptosis-related risk genes in glioblastoma (GBM) and drug sensitivity. Recent research suggests that LY-294002 is a potent and selective PI3K inhibitor that increases the chemosensitivity of liver cancer to oxaliplatin by blocking the PI3K/AKT/HIF-1α pathway (Xu et al., 2021). Tamoxifen is a selective estrogen receptor modulator, and it has been shown to increase oxidative stress and induce cell death by regulating reactive oxygen species (ROS), the accumulation of which leads to the production of lipid hydroperoxides. Activation of ferroptosis in tamoxifen-resistant breast cancer with high levels of fascin may serve as a potential treatment (Chen et al., 2022). Imatinib is an oral targeted therapy drug known as a tyrosine kinase inhibitor. Cysteine depletion in an imatinib-resistant chronic myeloid leukemia cell line induces ferroptosis (Mynott et al., 2023). However, the relationship between their effects on macrophage polarization and drug sensitivity is unclear, and the specific mechanism and *in vivo* manifestation need to be confirmed. Further studies are needed to understand the complex interplay between ferroptosis and M1/M2 macrophage polarization in gliomas and to develop effective therapeutic interventions.

However, there are several limitations to this study. Although data from the TCGA and CGGA databases were used to analyze and validate this study, further validation of the results using other databases and more clinical samples from different hospitals is needed. In addition, only eight ferroptosis-related genes were suggested to be associated with M2 macrophage polarization, and their specific mechanisms need to be further verified *in vivo* and *in vitro*. Also, we only examined the changes in the ratio of M1 and M2 macrophages in the samples without investigating other aspects such as their morphology. As mentioned above, M1 and M2 macrophages are morphologically different, and this has a significant impact on the polarization of TAMs, so we would like to further investigate the relationship between different macrophage morphologies and gliomas at a later stage. In addition, further *in vitro* and *in vivo* experiments on the relationship between the expression of various ferroptosis-related genes and drug sensitivity are needed to shed light on the clinical treatment of gliomas.

## 5 Conclusion

In conclusion, our results suggest that the expression of ferroptosis-related genes is associated with the prognosis of glioma patients and plays a potential role in M1 and M2 macrophage polarization. This may be a potential therapeutic target for glioma immunotherapy.

## Data availability statement

The datasets presented in this study can be found in online repositories. The names of the repository/repositories and accession number(s) can be found in the article/Supplementary material.

## Ethics statement

Ethical approval was not required for the studies on humans in accordance with the local legislation and institutional requirements because only commercially available established cell lines were used.

## Author contributions

XX: Data curation, Formal analysis, Methodology, Validation, Visualization, Writing – original draft. YZ: Data curation, Formal analysis, Methodology, Validation, Visualization, Writing – original draft. CL: Data curation, Methodology, Validation, Writing – original draft. HZ: Data curation, Methodology, Validation, Writing – original draft. YW: Conceptualization, Project administration, Supervision, Writing – review and editing. WZ: Conceptualization, Funding acquisition, Project administration, Supervision, Writing – review and editing.

## Footnotes

1
https://portal.gdc.cancer.gov

2
http://cgga.org.cn

3
http://www.broadinstitute.org/gsea/msigdb

4
http://xteam.xbio.top/CellMarker/index.jsp
