# Supplementary material for: Impact of ferroptosis-related risk genes on macrophage M1/M2 polarization and prognosis in glioblastoma
Source: Front Cell Neurosci. 2024 Jan 10;17:1294029. doi: 10.3389/fncel.2023.1294029 (PMC10817728; doi:10.3389/fncel.2023.1294029)
Supplement: Supplementary file 1 [file Table_1.DOCX]

Supplementary Material

# Supplementary Figures and Tables

## Supplementary Figures


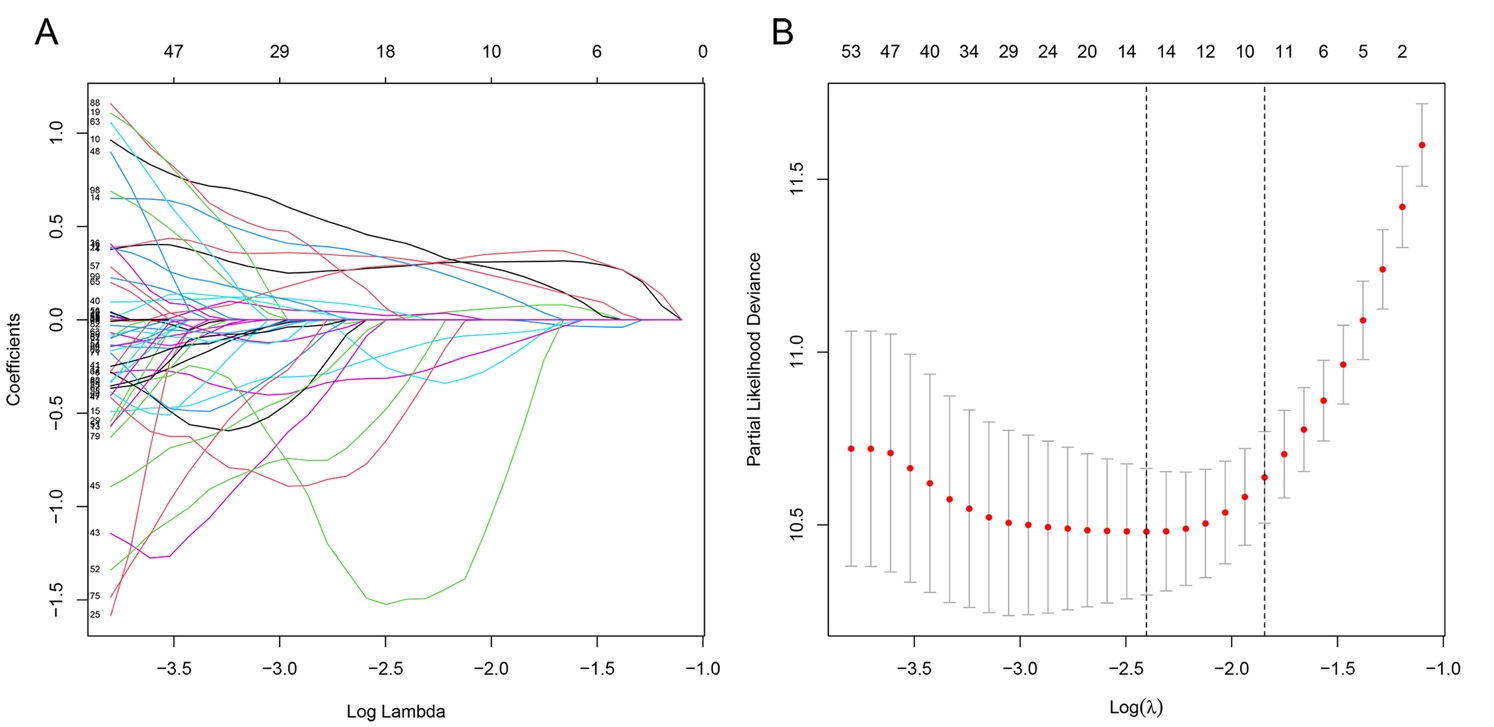


**Supplementary Figure 1.** Minimum criteria and coefficients of fourteen ferroptosis-related genes.


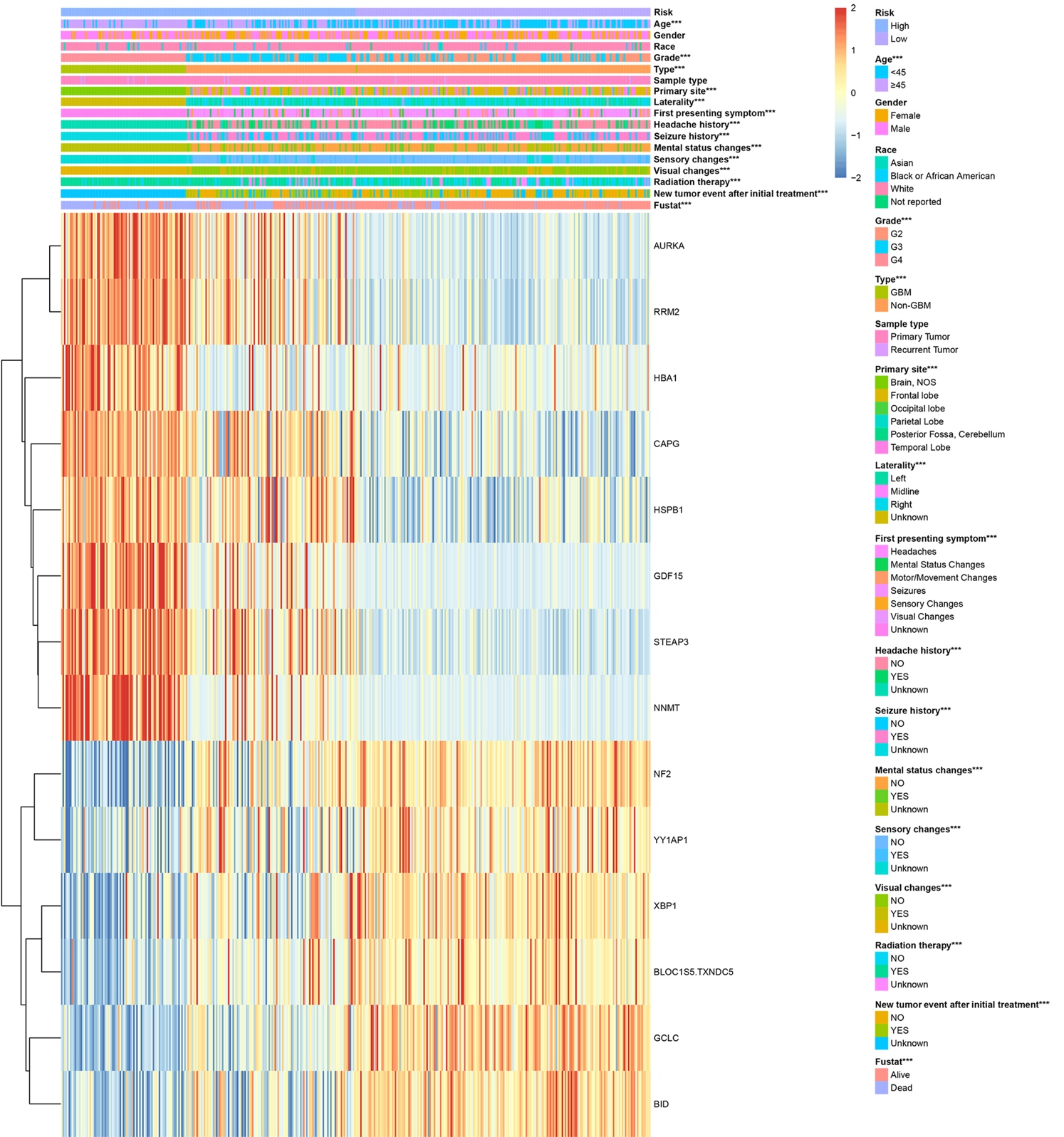


**Supplementary Figure 2.** The expression levels of fourteen ferroptosis-related risk genes with different clinicopathological characteristics and prognosis analysis in high and low risk glioma patients.


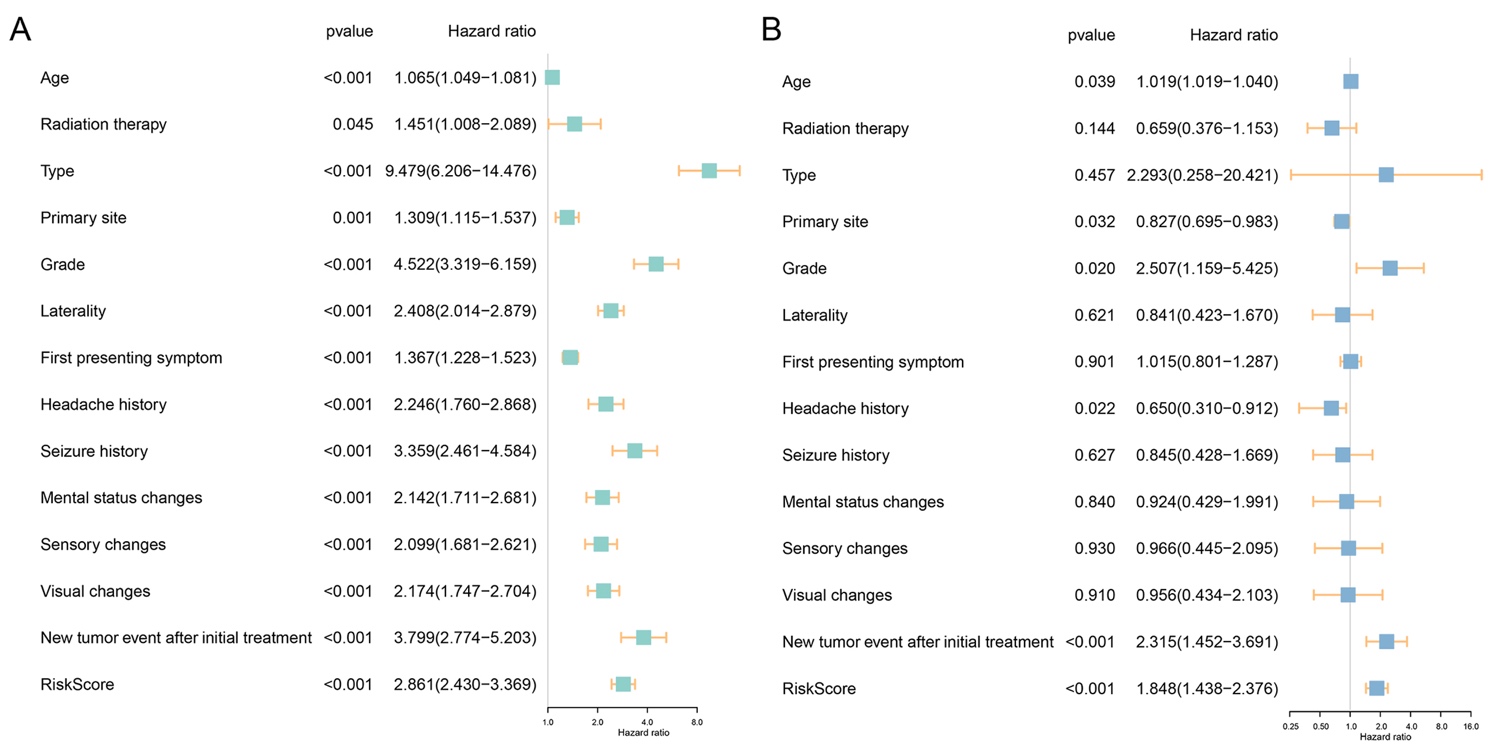


**Supplementary Figure 3.** Internal validation of independent prognostic risk factors in glioma patients conducted in the testing set of TCGA database. Univariate (A) and multivariate Cox regression analysis of clinicopathological characteristics (B) were performed, and the hazard ratios (HR) and 95% confidence intervals (CI) were calculated, respectively.


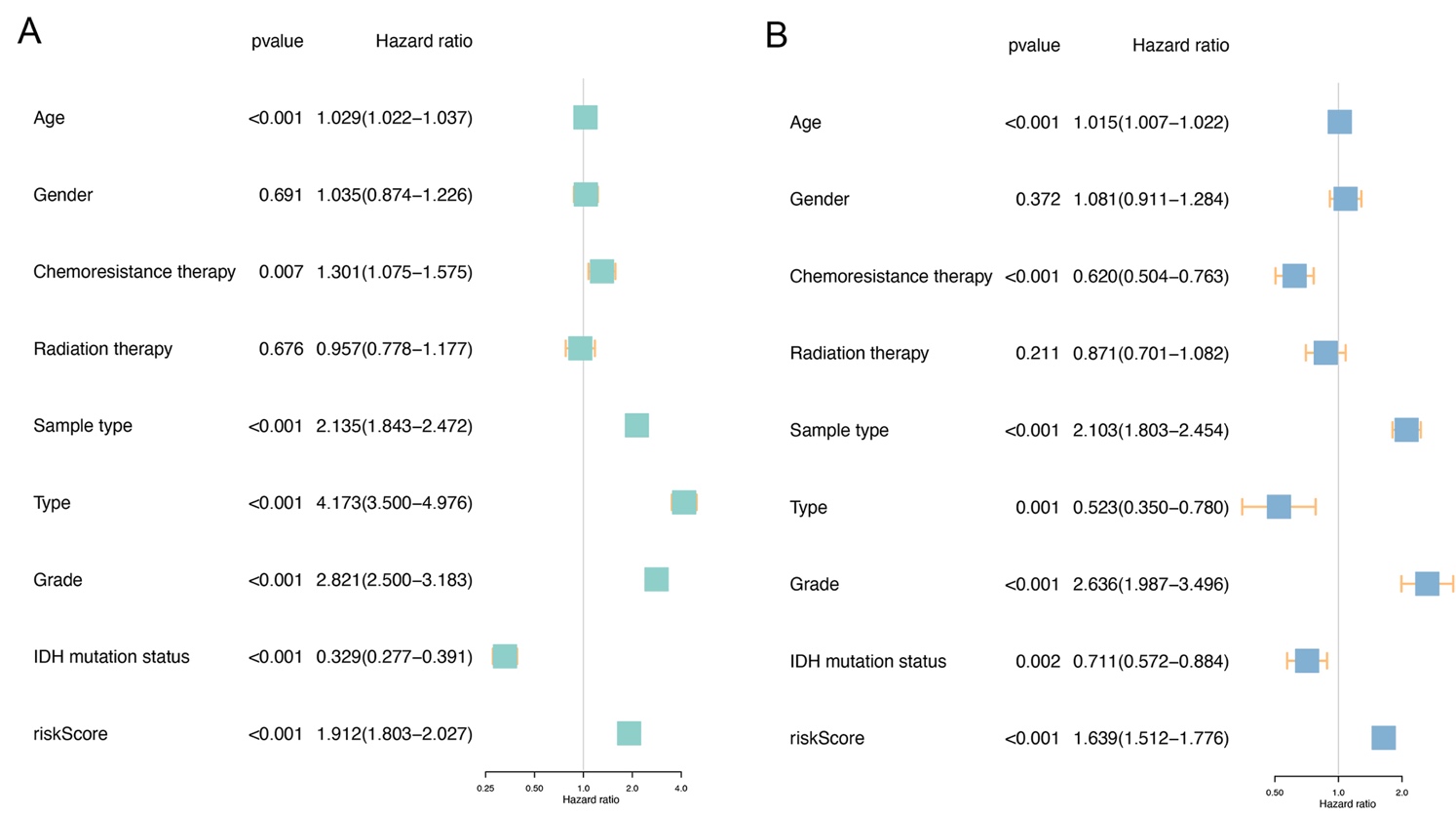


**Supplementary Figure 4.** Independent validation of independent prognostic risk factors in glioma patients conducted in CGGA database. Univariate (A) and multivariate Cox regression analysis of clinicopathological characteristics (B) were performed, and the hazard ratios (HR) and 95% confidence intervals (CI) were calculated, respectively.


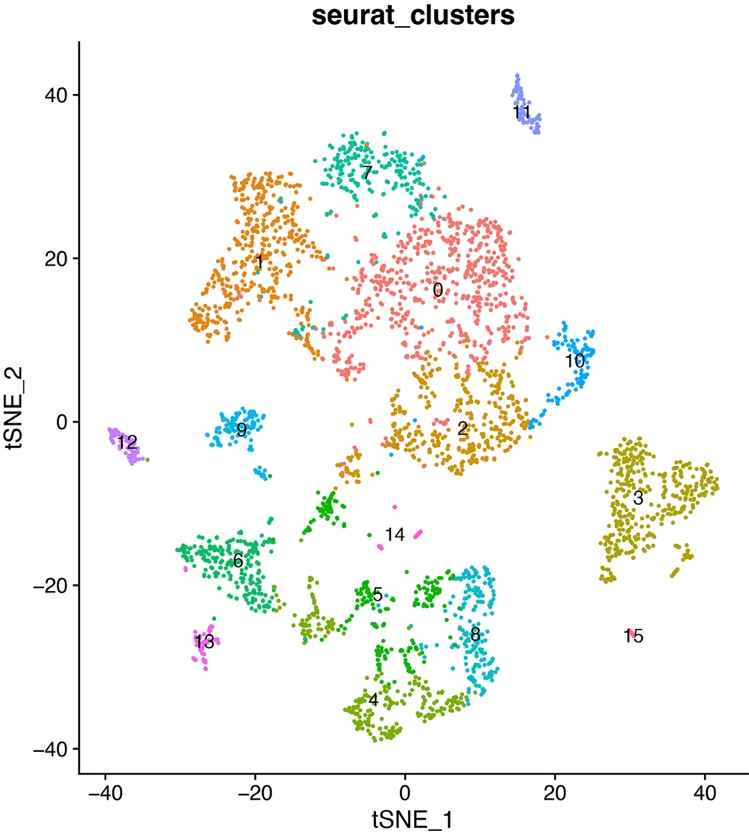


**Supplementary Figure 5.** The single cell cluster analysis in GBM from the GSE84465 database.

## Supplementary Tables

| **Gene** | **Ensemble ID** | **Description** | **Coefficient** |
| --- | --- | --- | --- |
| GDF15 | ENSG00000130513 | Growth differentiation factor 15 | 0.28917292792742 |
| STEAP3 | ENSG00000115107 | STEAP3 metalloreductase | 0.293571824229205 |
| NNMT | ENSG00000166741 | Nicotinamide N-methyltransferase | 0.0129497155850461 |
| NF2 | ENSG00000186575 | NF2, moesin-ezrin-radixin like (MERLIN) tumor suppressor | -0.291960993297964 |
| GCLC | ENSG00000001084 | Glutamate-cysteine ligase catalytic subunit | -0.298368095042284 |
| AURKA | ENSG00000087586 | Aurora kinase A | 0.409247230440991 |
| RRM2 | ENSG00000171848 | Ribonucleotide reductase regulatory subunit M2 | 0.324347297003548 |
| HSPB1 | ENSG00000106211 | Heat shock protein family B (small) member 1 | 0.302225199451812 |
| BID | ENSG00000015475 | BH3 interacting domain death agonist | -0.15485459467236 |
| CAPG | ENSG00000042493 | Capping actin protein, gelsolin like | 0.024808719973683 |
| XBP1 | ENSG00000100219 | X-box binding protein 1 | -1.49651505021173 |
| HBA1 | ENSG00000206172 | Hemoglobin subunit alpha 1 | 0.0297047204659537 |
| BLOC1S5 | ENSG00000188428 | Biogenesis of lysosomal organelles complex 1 subunit 5 | -0.491271287570775 |
| YY1AP1 | ENSG00000163374 | YY1 associated protein 1 | -0.34132529089156 |

**Supplementary Table 1.** Detailed description of related risk genes.

**Supplementary Table 2.** Macrophage markers obtained from previously published literature and the CellMarker website (http://xteam.xbio.top/CellMarker/index.jsp).

| **Gene** | **Cell Type** |
| --- | --- |
| ARG2 | Macrophage |
| BHLHE40 | Macrophage |
| CD74 | Macrophage |
| CD93 | Macrophage |
| CIB1 | Macrophage |
| CIITA | Macrophage |
| CREM | Macrophage |
| CYBB | Macrophage |
| CYTH1 | Macrophage |
| DOK3 | Macrophage |
| DSE | Macrophage |
| EMB | Macrophage |
| FAM49A | Macrophage |
| FGR | Macrophage |
| FOSL2 | Macrophage |
| FPR3 | Macrophage |
| FXYD5 | Macrophage |
| GPR132 | Macrophage |
| GPR65 | Macrophage |
| HLA-DMB | Macrophage |
| HLA-DQA1 | Macrophage |
| HLA-DRB5 | Macrophage |
| IFITM2 | Macrophage |
| IL10 | Macrophage |
| IL1RN | Macrophage |
| IQGAP1 | Macrophage |
| ITGA4 | Macrophage |
| KYNU | Macrophage |
| LYZ | Macrophage |
| METRNL | Macrophage |
| MS4A6A | Macrophage |
| MS4A7 | Macrophage |
| MXD1 | Macrophage |
| NFIL3 | Macrophage |
| PDE4B | Macrophage |
| PIM1 | Macrophage |
| PLAC8 | Macrophage |
| PLBD1 | Macrophage |
| PLTP | Macrophage |
| PQLC3 | Macrophage |
| PTPN7 | Macrophage |
| S100A11 | Macrophage |
| SAMHD1 | Macrophage |
| SH3BGRL | Macrophage |
| SPINT2 | Macrophage |
| SYNGR2 | Macrophage |
| TGFBI | Macrophage |
| THBD | Macrophage |
| TMEM123 | Macrophage |
| TNFSF13 | Macrophage |
| TREM1 | Macrophage |
| VOPP1 | Macrophage |
| CD80 | MacrophageM1 |
| CD86 | MacrophageM1 |
| CD64 | MacrophageM1 |
| CD32 | MacrophageM1 |
| CD68 | MacrophageM1 |
| CD45 | MacrophageM1 |
| CD11 | MacrophageM1 |
| MHC II | MacrophageM1 |
| IL-1R | MacrophageM1 |
| TLR2 | MacrophageM1 |
| TLR4 | MacrophageM1 |
| iNOS | MacrophageM1 |
| SOCS3 | MacrophageM1 |
| CD206 | MacrophageM2 |
| CD163 | MacrophageM2 |
| CD68 | MacrophageM2 |
| CD16 | MacrophageM2 |
| IL-10 | MacrophageM2 |
| MRC1 | MacrophageM2 |
| CXCR4 | MacrophageM2 |
| IL27RA | MacrophageM2 |
| CSF1R | MacrophageM2 |
| CCL2 | MacrophageM2 |
| CCL7 | MacrophageM2 |
| CCL17 | MacrophageM2 |
| CCL18 | MacrophageM2 |
| CCL23 | MacrophageM2 |
