# Supplementary material for: Impact of ferroptosis-related risk genes on macrophage M1/M2 polarization and prognosis in glioblastoma
Source: Front Cell Neurosci. 2024 Jan 10;17:1294029. doi: 10.3389/fncel.2023.1294029 (PMC10817728; doi:10.3389/fncel.2023.1294029)
Supplement: Supplementary file 3 [file Table_3.DOCX]

**Shared differentially expressed gene (DEG)**

| **id** | **HR** | **HR.95L** | **HR.95H** | **P value** |
| --- | --- | --- | --- | --- |
| ABCC1 | 1.80013983 | 1.00349644 | 3.22921266 | 0.04864724 |
| ACO1 | 0.04487939 | 0.02010006 | 0.10020666 | <0.0001 |
| ACSF2 | 1.33786991 | 0.73275071 | 2.44270786 | 0.34331481 |
| ACSL3 | 0.44318858 | 0.24184256 | 0.81216522 | 0.00845892 |
| ACSL4 | 0.12007336 | 0.06090358 | 0.23672847 | <0.0001 |
| ACVR1B | 0.19139391 | 0.09540338 | 0.38396573 | <0.0001 |
| AGPAT3 | 0.08907687 | 0.0434136 | 0.18276966 | <0.0001 |
| AIFM2 | 0.31203313 | 0.12814106 | 0.75982417 | 0.01032188 |
| AKR1C1 | 0.17961099 | 0.1028779 | 0.31357667 | <0.0001 |
| AKR1C2 | 0.16619175 | 0.05226411 | 0.52846397 | 0.00236167 |
| AKR1C3 | 0.22815694 | 0.16411276 | 0.31719404 | <0.0001 |
| ALB | 0.0019385 | 0.00039232 | 0.00957842 | <0.0001 |
| ALOX12 | 0.02265356 | 0.00547755 | 0.09368858 | <0.0001 |
| ALOX12B | 0.00843915 | 0.00190586 | 0.03736857 | <0.0001 |
| ALOX15 | 0.01096924 | 0.00103301 | 0.116479 | 0.00018141 |
| ALOX15B | 1.20240592 | 0.8680167 | 1.6656131 | 0.26758796 |
| ALOX5 | 2.77621837 | 1.93265344 | 3.98798267 | <0.0001 |
| ALOXE3 | 0.00029602 | 3.91E-05 | 0.0022427 | <0.0001 |
| ANGPTL7 | 0.00049895 | 4.00E-05 | 0.00622099 | <0.0001 |
| ANO6 | 2.80862555 | 1.75377227 | 4.49794856 | <0.0001 |
| ARRDC3 | 6.80809547 | 3.76997266 | 12.2945623 | <0.0001 |
| ASNS | 2.12721061 | 1.21093877 | 3.73679093 | 0.00864516 |
| ATF3 | 2.82107848 | 2.13595854 | 3.72595424 | <0.0001 |
| ATF4 | 0.19258355 | 0.08483396 | 0.43718837 | <0.0001 |
| ATG13 | 0.02795564 | 0.01346022 | 0.05806127 | <0.0001 |
| ATG16L1 | 1.85125316 | 0.55426258 | 6.18323952 | 0.31687356 |
| ATG3 | 0.11742161 | 0.02492929 | 0.55307777 | 0.00674864 |
| ATG4D | 0.78099074 | 0.32687818 | 1.86597509 | 0.57803437 |
| ATG5 | 1.75646027 | 0.4718917 | 6.53784048 | 0.40089494 |
| ATG7 | 0.30593646 | 0.07431123 | 1.25952857 | 0.10092457 |
| ATM | 0.05793839 | 0.02714669 | 0.1236562 | <0.0001 |
| ATP5MC3 | 3.65303431 | 1.82105067 | 7.3280002 | 0.00026472 |
| ATP6V1G2 | 0.30247983 | 0.24034173 | 0.38068315 | <0.0001 |
| AURKA | 6.68143786 | 4.66496398 | 9.56955125 | <0.0001 |
| BACH1 | 0.81656777 | 0.35009827 | 1.90455937 | 0.63908367 |
| BAP1 | 0.14259866 | 0.0672145 | 0.30252962 | <0.0001 |
| BECN1 | 0.0568867 | 0.02065945 | 0.15664002 | <0.0001 |
| BID | 0.10911024 | 0.07046526 | 0.16894912 | <0.0001 |
| BLOC1S5.TXNDC5 | 0.00015998 | 1.72E-05 | 0.00148414 | <0.0001 |
| BNIP3 | 0.2781262 | 0.14450825 | 0.53529253 | 0.00012776 |
| BRD4 | 0.64249762 | 0.24009934 | 1.71930159 | 0.37837538 |
| CA9 | 2.64731947 | 2.15983091 | 3.2448375 | <0.0001 |
| CAPG | 4.34191436 | 3.22642752 | 5.84306332 | <0.0001 |
| CARS1 | 0.32170247 | 0.12637684 | 0.81891968 | 0.01735914 |
| CAV1 | 2.33243647 | 1.81340799 | 3.0000198 | <0.0001 |
| CBS | 0.14414963 | 0.06953582 | 0.29882607 | <0.0001 |
| CD44 | 2.59548974 | 1.94992887 | 3.45477577 | <0.0001 |
| CDKN1A | 1.79545551 | 1.37198373 | 2.34963463 | <0.0001 |
| CDKN2A | 1.07930541 | 0.82130915 | 1.41834556 | 0.58399124 |
| CDO1 | 1.32551097 | 0.79342889 | 2.21441312 | 0.28182077 |
| CEBPG | 2.30068833 | 1.08775271 | 4.866149 | 0.02925426 |
| CHAC1 | 1.96796888 | 1.2146234 | 3.18856159 | 0.00596564 |
| CHMP5 | 0.11175673 | 0.04475166 | 0.27908611 | <0.0001 |
| CHMP6 | 0.12454939 | 0.04789049 | 0.3239171 | <0.0001 |
| CISD1 | 0.07093288 | 0.03693881 | 0.13621104 | <0.0001 |
| CISD2 | 19.9206724 | 7.39215701 | 53.6830033 | <0.0001 |
| CS | 0.115851 | 0.05000322 | 0.26841177 | <0.0001 |
| CXCL2 | 0.84889755 | 0.58741381 | 1.2267792 | 0.38321319 |
| CYBB | 1.32411081 | 0.9880507 | 1.77447315 | 0.06017831 |
| DDIT3 | 2.4230199 | 1.80339546 | 3.25553965 | <0.0001 |
| DDIT4 | 0.41072241 | 0.30865972 | 0.54653355 | <0.0001 |
| DNAJB6 | 9.37727692 | 3.53183901 | 24.897319 | <0.0001 |
| DPP4 | 3.56363811 | 2.49084548 | 5.09847625 | <0.0001 |
| DRD4 | 0.18696852 | 0.09087328 | 0.38468102 | <0.0001 |
| DRD5 | 0.00844968 | 0.00162388 | 0.04396696 | <0.0001 |
| DUOX1 | 0.00478467 | 0.00137763 | 0.0166177 | <0.0001 |
| DUOX2 | 7.27E-05 | 6.37E-06 | 0.00082862 | <0.0001 |
| DUSP1 | 1.39585641 | 1.02809205 | 1.89517575 | 0.03255493 |
| EGFR | 1.64789392 | 1.32592918 | 2.04803877 | <0.0001 |
| EGLN2 | 1.80076833 | 0.79309061 | 4.08877186 | 0.15975555 |
| EIF2AK4 | 1.80369951 | 0.67424824 | 4.82512484 | 0.24004813 |
| EIF2S1 | 0.4649206 | 0.19774788 | 1.09306439 | 0.07909723 |
| ELAVL1 | 0.82819429 | 0.28814676 | 2.38040424 | 0.72637731 |
| EMC2 | 0.02804895 | 0.01337104 | 0.05883937 | <0.0001 |
| ENPP2 | 0.80069323 | 0.61738019 | 1.03843573 | 0.09380779 |
| EPAS1 | 0.45205406 | 0.28179916 | 0.72517206 | 0.00099255 |
| FADS2 | 0.23300444 | 0.15897624 | 0.3415043 | <0.0001 |
| FANCD2 | 4.44295822 | 2.90557082 | 6.79380367 | <0.0001 |
| FBXW7 | 0.51731319 | 0.29832405 | 0.89705452 | 0.01893662 |
| FH | 2.52241004 | 0.96540466 | 6.5905549 | 0.05901064 |
| FLT3 | 0.00077785 | 0.00011558 | 0.00523474 | <0.0001 |
| FTH1 | 5.34846027 | 2.60027621 | 11.0011495 | <0.0001 |
| FTL | 5.41406714 | 3.51860479 | 8.33060963 | <0.0001 |
| FTMT | 9.19E-06 | 6.97E-07 | 0.00012109 | <0.0001 |
| G6PD | 12.4118417 | 5.77698817 | 26.6668044 | <0.0001 |
| GABARAPL1 | 0.10865743 | 0.06357262 | 0.18571574 | <0.0001 |
| GABARAPL2 | 0.07095047 | 0.03726716 | 0.13507787 | <0.0001 |
| GABPB1 | 0.05787975 | 0.01909934 | 0.17540218 | <0.0001 |
| GCH1 | 3.06535703 | 1.94269684 | 4.83678848 | <0.0001 |
| GCLC | 0.05967384 | 0.03534933 | 0.10073649 | <0.0001 |
| GDF15 | 4.65915875 | 3.66462442 | 5.92359754 | <0.0001 |
| GLS2 | 0.00087133 | 0.00011114 | 0.00683099 | <0.0001 |
| GOT1 | 0.37674955 | 0.23272273 | 0.60991132 | <0.0001 |
| GPT2 | 0.11778761 | 0.0744562 | 0.18633667 | <0.0001 |
| GPX2 | 5.47E-05 | 4.90E-06 | 0.0006104 | <0.0001 |
| GPX4 | 0.59616863 | 0.25089623 | 1.41658982 | 0.24147174 |
| HAMP | 2.1138338 | 1.67125112 | 2.67362174 | <0.0001 |
| HBA1 | 2.86093696 | 2.21570358 | 3.69406826 | <0.0001 |
| HELLS | 2.83130787 | 1.56947961 | 5.10761927 | 0.00054554 |
| HERPUD1 | 0.42394467 | 0.17482115 | 1.02807406 | 0.05760332 |
| HIC1 | 3.57108667 | 1.16229135 | 10.9719994 | 0.02624502 |
| HIF1A | 0.87701294 | 0.53945086 | 1.42580496 | 0.59661301 |
| HILPDA | 3.71359264 | 2.82686092 | 4.87847499 | <0.0001 |
| HMGB1 | 0.33392131 | 0.14279661 | 0.78085492 | 0.01138363 |
| HMOX1 | 3.62924285 | 2.72083207 | 4.84094693 | <0.0001 |
| HNF4A | 7.57E-06 | 6.30E-07 | 9.09E-05 | <0.0001 |
| HRAS | 0.46558648 | 0.24340587 | 0.89057328 | 0.02087808 |
| HSD17B11 | 2.05255305 | 1.00262447 | 4.20194614 | 0.04916775 |
| HSF1 | 0.16920657 | 0.07062979 | 0.40536526 | <0.0001 |
| HSPA5 | 4.82707181 | 2.50797486 | 9.29061235 | <0.0001 |
| HSPB1 | 5.26837163 | 3.80039978 | 7.30337366 | <0.0001 |
| IDH1 | 5.83798532 | 3.1702492 | 10.7505973 | <0.0001 |
| IFNG | 0.00175344 | 0.00012732 | 0.02414819 | <0.0001 |
| IL33 | 0.57728441 | 0.41047858 | 0.81187498 | 0.00158967 |
| IL6 | 2.54883155 | 1.79654029 | 3.6161406 | <0.0001 |
| IREB2 | 0.15502293 | 0.06130969 | 0.39197899 | <0.0001 |
| ISCU | 0.23317457 | 0.09546822 | 0.56951289 | 0.00139544 |
| JDP2 | 0.44111626 | 0.18638485 | 1.04398805 | 0.06259935 |
| JUN | 1.97156204 | 1.37563841 | 2.8256385 | 0.00021842 |
| KEAP1 | 0.03412998 | 0.01279554 | 0.09103602 | <0.0001 |
| KLHL24 | 0.06335579 | 0.02948486 | 0.13613619 | <0.0001 |
| KRAS | 0.07709635 | 0.0322967 | 0.18403883 | <0.0001 |
| LAMP2 | 1.53769198 | 0.74730697 | 3.16402324 | 0.24249712 |
| LONP1 | 0.0798697 | 0.03510607 | 0.18171128 | <0.0001 |
| LPCAT3 | 3.1556922 | 1.48914008 | 6.68734487 | 0.00270714 |
| LPIN1 | 0.07720563 | 0.04556078 | 0.13082985 | <0.0001 |
| LURAP1L | 0.64505175 | 0.32008288 | 1.29995006 | 0.22010392 |
| MAFG | 0.12212457 | 0.05758731 | 0.25898777 | <0.0001 |
| MAP1LC3A | 1.88836043 | 1.35668209 | 2.62840141 | 0.00016453 |
| MAP3K5 | 0.18104105 | 0.12620502 | 0.2597033 | <0.0001 |
| MAPK1 | 0.07289686 | 0.03927955 | 0.13528549 | <0.0001 |
| MAPK14 | 0.6781375 | 0.22588797 | 2.03583426 | 0.48863057 |
| MAPK3 | 0.04389488 | 0.02039931 | 0.09445223 | <0.0001 |
| MAPK8 | 0.04458833 | 0.02527545 | 0.07865811 | <0.0001 |
| MAPK9 | 0.03416067 | 0.01433092 | 0.08142889 | <0.0001 |
| MIOX | 5.60E-05 | 3.50E-06 | 0.00089527 | <0.0001 |
| MT1G | 1.50142501 | 1.15267709 | 1.95568826 | 0.0025823 |
| MT3 | 1.74767482 | 1.33174288 | 2.29351123 | <0.0001 |
| MTDH | 0.57356734 | 0.24450545 | 1.34548944 | 0.20131826 |
| MTOR | 0.27751731 | 0.13595974 | 0.56646078 | 0.00042969 |
| MUC1 | 3.43993853 | 2.13489331 | 5.54274869 | <0.0001 |
| MYB | 1.7951829 | 0.60141301 | 5.3585167 | 0.29433692 |
| NCF2 | 4.24336419 | 2.62508105 | 6.85926998 | <0.0001 |
| NCOA4 | 0.03928827 | 0.0209765 | 0.07358558 | <0.0001 |
| NF2 | 0.02996091 | 0.01565255 | 0.05734889 | <0.0001 |
| NFE2L2 | 1.65919262 | 0.64097315 | 4.29490715 | 0.29675615 |
| NFS1 | 0.03473322 | 0.01234391 | 0.09773208 | <0.0001 |
| NGB | 0.59395826 | 0.38508178 | 0.91613376 | 0.01846656 |
| NNMT | 2.57601926 | 2.19895267 | 3.01774354 | <0.0001 |
| NOS2 | 1.45692996 | 1.07650226 | 1.9717979 | 0.01479277 |
| NOX1 | 0.00778956 | 0.00164279 | 0.03693539 | <0.0001 |
| NOX3 | 0.00095191 | 0.00010408 | 0.00870582 | <0.0001 |
| NOX4 | 16.2347414 | 8.70682962 | 30.2712744 | <0.0001 |
| NOX5 | 1.09E-05 | 8.93E-07 | 0.00013252 | <0.0001 |
| NQO1 | 3.16597779 | 2.07888595 | 4.82153209 | <0.0001 |
| NRAS | 6.48289578 | 3.2151958 | 13.0716573 | <0.0001 |
| OTUB1 | 0.05716099 | 0.02309439 | 0.14147935 | <0.0001 |
| OXSR1 | 0.40441869 | 0.14369063 | 1.13824039 | 0.08639791 |
| PANX1 | 0.59912886 | 0.3179866 | 1.12883812 | 0.11296576 |
| PCK2 | 2.64398843 | 1.45236873 | 4.81329204 | 0.00146821 |
| PEBP1 | 0.23475278 | 0.15334057 | 0.3593887 | <0.0001 |
| PGD | 2.51095305 | 1.21189276 | 5.20251086 | 0.01324804 |
| PHKG2 | 0.09651395 | 0.02754969 | 0.33811424 | 0.00025697 |
| PIK3CA | 0.11213331 | 0.05317671 | 0.2364546 | <0.0001 |
| PLIN2 | 4.57072725 | 3.31198561 | 6.30786183 | <0.0001 |
| PLIN4 | 0.20564651 | 0.07271173 | 0.58161856 | 0.00286716 |
| PML | 1.64378424 | 0.58820843 | 4.59365511 | 0.34319471 |
| PRDX1 | 1.76969319 | 1.19151753 | 2.62842461 | 0.00468163 |
| PRDX6 | 2.02215919 | 1.25817404 | 3.25004939 | 0.0036306 |
| PRKAA1 | 3.21316242 | 1.08600433 | 9.50678781 | 0.03494137 |
| PRKAA2 | 0.0546547 | 0.0195993 | 0.15241037 | <0.0001 |
| PROM2 | 0.00454527 | 0.00100811 | 0.02049332 | <0.0001 |
| PSAT1 | 0.23597558 | 0.15860985 | 0.3510783 | <0.0001 |
| PTGS2 | 1.95673569 | 1.23975612 | 3.08836108 | 0.00393947 |
| RB1 | 2.04305337 | 1.0054257 | 4.15154205 | 0.04827705 |
| RELA | 0.26876962 | 0.10892133 | 0.66320443 | 0.00435679 |
| RGS4 | 0.80693313 | 0.64974186 | 1.00215351 | 0.05231844 |
| RIPK1 | 31.8766976 | 12.7652535 | 79.6007581 | <0.0001 |
| RPL8 | 0.367218 | 0.21430146 | 0.62924938 | 0.00026663 |
| RRM2 | 3.55951762 | 2.78510768 | 4.54925523 | <0.0001 |
| SAT1 | 5.00026713 | 3.31372178 | 7.54519332 | <0.0001 |
| SCD | 0.2056988 | 0.15559553 | 0.27193581 | <0.0001 |
| SCP2 | 1.28540469 | 0.62653763 | 2.63713643 | 0.493484 |
| SELENOS | 2.9630854 | 1.15774306 | 7.58361283 | 0.02348523 |
| SESN2 | 1.23062228 | 0.57498367 | 2.63386818 | 0.59298416 |
| SETD1B | 0.11661573 | 0.05364316 | 0.25351283 | <0.0001 |
| SIRT1 | 0.03828292 | 0.02068324 | 0.07085844 | <0.0001 |
| SLC1A4 | 0.173948 | 0.12063765 | 0.25081645 | <0.0001 |
| SLC1A5 | 2.11534447 | 1.47753914 | 3.02846951 | <0.0001 |
| SLC2A1 | 1.67798232 | 0.93324558 | 3.01702438 | 0.08378046 |
| SLC2A12 | 0.26108316 | 0.10466939 | 0.65123542 | 0.00398152 |
| SLC2A14 | 0.00038641 | 2.78E-05 | 0.00536922 | <0.0001 |
| SLC2A3 | 2.920048 | 2.02362129 | 4.21357513 | <0.0001 |
| SLC2A6 | 0.19053403 | 0.0936548 | 0.38762793 | <0.0001 |
| SLC2A8 | 0.09311194 | 0.02759635 | 0.31416593 | 0.00013025 |
| SLC38A1 | 0.28141142 | 0.21109756 | 0.37514593 | <0.0001 |
| SLC3A2 | 1.06209817 | 0.47778733 | 2.36099294 | 0.88248802 |
| SLC40A1 | 2.49026424 | 1.63750095 | 3.78712205 | <0.0001 |
| SLC7A11 | 0.50185575 | 0.33324762 | 0.75577194 | 0.00096539 |
| SLC7A5 | 0.26493407 | 0.14941776 | 0.46975714 | <0.0001 |
| SNX4 | 1.85556245 | 0.63871816 | 5.3906593 | 0.25591543 |
| SOCS1 | 6.81103684 | 4.63112252 | 10.017058 | <0.0001 |
| SP1 | 0.37655144 | 0.17393807 | 0.81518091 | 0.01319288 |
| SQSTM1 | 4.61868129 | 1.9346004 | 11.0266786 | 0.00056842 |
| SRC | 0.11703337 | 0.06138131 | 0.22314299 | <0.0001 |
| SRXN1 | 0.60421545 | 0.14640359 | 2.49362959 | 0.4860519 |
| STAT3 | 5.85873452 | 2.46865043 | 13.9042652 | <0.0001 |
| STEAP3 | 4.6160819 | 3.58551733 | 5.9428557 | <0.0001 |
| STMN1 | 0.42745126 | 0.25723157 | 0.71031164 | 0.001038 |
| TF | 0.5395255 | 0.43501271 | 0.66914773 | <0.0001 |
| TFAP2C | 0.43163494 | 0.1221689 | 1.52500948 | 0.19200791 |
| TFR2 | 3.89048379 | 2.25047217 | 6.72563933 | <0.0001 |
| TFRC | 7.09984095 | 4.70694774 | 10.7092206 | <0.0001 |
| TGFBR1 | 2.60447646 | 1.55655258 | 4.35789816 | 0.0002677 |
| TLR4 | 0.49847019 | 0.35144449 | 0.70700362 | <0.0001 |
| TMBIM4 | 4.92488755 | 2.2319204 | 10.8671068 | <0.0001 |
| TNFAIP3 | 5.00070944 | 2.97101268 | 8.41702731 | <0.0001 |
| TP53 | 1.99324348 | 1.25890827 | 3.15592458 | 0.00326081 |
| TP63 | 2.37049653 | 0.93656757 | 5.99983811 | 0.0685082 |
| TRIB3 | 2.28399206 | 1.55910731 | 3.34590164 | <0.0001 |
| TSC22D3 | 0.5686268 | 0.35848933 | 0.90194159 | 0.01646518 |
| TUBE1 | 0.20311523 | 0.09150433 | 0.45086168 | <0.0001 |
| TXNIP | 0.41178517 | 0.2598648 | 0.65252019 | 0.00015834 |
| TXNRD1 | 2.12742996 | 0.84694388 | 5.3438703 | 0.10817315 |
| UBC | 0.56002519 | 0.16721433 | 1.87560602 | 0.34715298 |
| ULK1 | 0.17653791 | 0.08417248 | 0.37025916 | <0.0001 |
| ULK2 | 0.20056608 | 0.1165521 | 0.34513966 | <0.0001 |
| VDAC2 | 0.07919393 | 0.0371936 | 0.16862252 | <0.0001 |
| VEGFA | 3.1855894 | 2.58838028 | 3.92059077 | <0.0001 |
| VLDLR | 0.87182317 | 0.46522875 | 1.63376757 | 0.66860816 |
| WIPI1 | 12.9223213 | 6.76251915 | 24.6929266 | <0.0001 |
| WIPI2 | 0.19844963 | 0.06373822 | 0.61787504 | 0.00525723 |
| XBP1 | 8.61E-06 | 6.78E-07 | 0.00010945 | <0.0001 |
| YWHAE | 0.07790355 | 0.03622893 | 0.16751703 | <0.0001 |
| YY1AP1 | 0.01350826 | 0.00448012 | 0.04072957 | <0.0001 |
| ZEB1 | 0.18464443 | 0.1260883 | 0.27039436 | <0.0001 |
| ZFP36 | 1.73419932 | 1.33598405 | 2.25111017 | <0.0001 |
| ZFP69B | 0.14616815 | 0.05717458 | 0.37368233 | <0.0001 |
| ZNF419 | 1.27828565 | 0.55607628 | 2.93847136 | 0.56318269 |
